# Disaster-induced migration and local labor markets: Heterogeneous effects by skill level and industry structure

**DOI:** 10.1371/journal.pone.0346313

**Published:** 2026-10-27

**Authors:** Yuchen Hu, Yihua Mao, Qiaoyan Luo, Harvey Cutler

**Affiliations:** 1 School of Emergency Management, Wuxi University, Wuxi, China; 2 Department of Economics, Colorado State University, Fort Collins, Colorado, United States of America; 3 Binhai Industrial Technology Research Institute of Zhejiang University, Tianjin, China; 4 School of Digital Economics and Management, Wuxi University, Wuxi, China; North Carolina State University, UNITED STATES OF AMERICA

## Abstract

Natural disasters can trigger large-scale population displacement, resulting in labor market shocks in destination areas. Nonetheless, important aspects of migration-driven shocks remain unclear, particularly how relative inflow intensity, the spatially concentrated inflow of low-skilled migrants, and industry structure moderate these impacts. Using Hurricane Katrina as a natural experiment and a Difference in Difference framework, this study reveals that displacement shock reduces wages in the low-skill localized sector, but this negative impact is heavily characterized by non-linear congestion effects, intensifying sharply from $-0.15 to $-0.27 drop in wage rates when the inflow concentration crosses certain threshold. Decomposing the demand-side channels shows that while the influx stimulates local business creation, this countervailing buffer is economically modest and insufficient to offset the supply-driven wage depression. Furthermore, sectoral mobility and joint wage-setting analyses confirm that low-skill localized and traded industries remain highly segmented in the short run. These findings highlight the importance of accounting for both supply and demand channels, non-linear thresholds, and sectoral frictions to improve regional labor market policies after demographic shocks.

## 1. Introduction

On Aug 29th, 2005, Hurricane Katrina made landfall on the gulf coast, causing a large number of displacements that ranged from 0.7 to 1 million [1–3]. Two years later, the population of the city of New Orleans was still about 70% of its pre-Katrina level [4]. The slow recovery of population indicated the evacuation may have turned to long-term migration, which could influence the labor market of the destination area.

The impact of migration on destination’s labor market has long been a subject of academic attention. Early studies focused on migrants’ self-selection and the associated impacts [5,6]. Recently, research has shifted toward examining labor market responses to external shocks such as natural disasters and wars [7–13]. Such studies create quasi-experimental settings to avoid endogeneity problems like reverse causality.

In fact, existing research has examined labor supply shocks following Hurricane Katrina [1,14]. Nonetheless, immigration affects labor markets in many ways. Significant gaps remain in our understanding of how migration-driven shocks affect labor markets, particularly along three critical dimensions below.

First, how does the relative intensity, rather than the absolute scale of in-migration, shape labor market outcomes. Prior research typically emphasizes destinations with the highest absolute number of migrants (e.g., Houston after Katrina), but overlooks smaller regions that may experience more substantial per capita shocks [15].

Second, as demonstrated by De Koning and Filatova [16], low-income migrants often remain within or relocate to nearby high-risk areas due to socioeconomic constraints. This spatial clustering may further depressing wages among low-income population. Studies suggested that for the Katrina evacuees who were less likely to return, they tended to be young and less educated [8,17,18]. If they stayed, the magnitude of the supply shock would be disproportionally larger for the low-skilled workers than for the high-skilled workers [19].

Third, to what extent do industry-specific structures moderate the labor market impacts of migration shocks. Trade-exposed industries are influenced mainly by global markets and supply-side factors, while local service sectors, such as food services and healthcare, are heavily dependent on local demand [20,21]. Except for the supply shocks, the sudden rise in the population could drive up demand for localized goods and services [17]. The interaction between consumption demand and supply shock lacks empirical quantification.

To test the above hypotheses, we first use the Difference in Difference (DID) approach where we compare the hourly wage rates in areas that received these evacuees to areas that didn’t in four separate markets. These markets consist of traded low-skilled, traded high-skilled, localized low-skilled and localized high-skilled. We find that among the four markets, localized low-skilled market experienced a statistically significant decline in wage rates, and this effect is non-linear in terms of the shock intensity.

An important empirical concern with documenting the above relationship is that, areas where the evacuees decided to settle in greater numbers, could have been on a different trajectory in terms of their wage rates prior to Hurricane Katrina. To handle this, we include a series of potentially important characteristics related both to the labor supply and the migration decision along with flexible year effects and interaction terms. Our design also incorporates non-employer firm establishment data to proxy localized demand, and we find slight offsetting effect from the demand side.

The rest of the paper is organized as follows. Section 2 provides the background information for Hurricane Katrina accompanied by the theoretical analyses for such inflow. Section 3 describes the data and empirical strategy. Section 4 discusses the results and Section 5 makes further analysis. Section 6 concludes and discusses the results.

## 2. The background of Hurricane Katrina and theoretical basis

Katrina made landfall on August 29^th^, 2005 near Buras, Louisiana, and led to catastrophic damage in Alabama, Louisiana, and Mississippi. There were roughly 138 counties and parishes impacted by the storm with an area of 93,000 miles [22]. The estimated damage reached around $96 billion and the number of casualties reached more than 1000 [22–24]. The huge waves brought by the wind damaged the levees and the floodwalls in New Orleans, so the subsequent flooding covered hundreds of thousands of homes in the Gulf Coasts area with huge displacement. It was estimated that 80% of the city of New Orleans was flooded and the entire population in the city was forced to evacuate [25].

With estimates ranging from 0.7 million to over 1 million on the volume of the displaced people, the story of recovery was not encouraging. Among those evacuees, nearly 50 percent of them came from New Orleans, a place with a high level of poverty and a negative growth rate of employment. Displaced residents were first allowed to return to the city of New Orleans at the end of September 2005 [26]. By October 2005 (approximately two months after the storm), 53 percent of evacuees had returned to their pre-Katrina counties [18]. About 13 months after the storm, slightly more than 50 percent returned to New Orleans. This number is similar across other data sources (e.g., the Current Population Survey (CPS), American Community Survey (ACS), Displaced New Orleans Residents Pilot Survey (DNRPS) and Survey of Low-income parents who attend two Community Colleges in New Orleans [27].

Studies have suggested that among the people who did not return in a year, they may never return over the longer terms. For example, two years after Katrina, the population of the city of New Orleans was about 70% of its pre-Katrina level [4]. Hence, we have a reason to believe that those people who didn’t return may have decided to relocate in the area they were evacuated to, and as a result, cause an increase in the supply of labor in those areas.

Basic economic theory for the market of labor predicts that migration can shift the supply curve to the right and results in a ceteris paribus decrease in the equilibrium wage rate. This comes from the neo-classical economic theory of diminishing marginal product of labor and the inelastic supply of labor.

While pursuing empirical evidence about the impact of immigration on labor markets, most studies recognized that the local labor markets are heterogenous with skill sets [28]. The leading economic scholar of immigration—George Borjas, suggested in Borjas [29] that the skills of the workers in terms of both education and work experience can help to identify the group of native workers that the immigrants would be competing with. In other words, downward pressure on wages will be the greatest for the markets in which the individuals’ skills are most like those of immigrants.

For Katrina, studies found that people who are less likely to return, they tend to be younger and less educated. Groen and Polivka [18] suggested that nearly 22 percent of the adult evacuees who did not return didn’t have a high school diploma, and about 53 percent of the adult evacuees who did not return were less than 40 years old. Therefore, we would expect a larger supply shock for the market of less-skilled workers.

However, the magnitude of the downward pressure on wages also depends on the steepness of the labor demand curve. All else equal, an elastic labor demand curve would generate a smaller fall in wages than inelastic labor demand, given the same magnitude of labor supply increases. Empirical studies did find that the labor demand for low-skilled workers is more elastic than for high-skilled workers. For instance, Hamermesh [30] reviewed about 15 papers that looked at the U.S. manufacturing sectors’ demand on labor and found that in most studies, demand elasticity is larger for low-skilled workers than high-skilled workers in absolute term. Freier and Steiner [31] found that the labor demand elasticity for unskilled labor tended to be higher than the skilled labor, in absolute terms, using information from 25 industries between 1999 and 2003 in West Germany. Using data for nine Western European countries between 1990 and 2004 on over 20 industries, Crinò [32] estimated the absolute value of the own-wage elasticity of labor demand for the low-skilled workers ranged from 0.67 to 1, while this number only ranged from 0.02 to 0.1 for high-skilled workers. A meta-analysis done by Lichter et al. [33] looked at over 100 studies in labor demand also concluded that low-skill worker is associated with a more sensitive demand curve. Hamermesh [30] explained this phenomenon as the skill ties, implying that the greater amount of human capital embodied by the high-skilled workers made the labor demand less sensitive to changes in wage rates.

Migration not only brought a large pool of potential workers, but also brought a substantial demand for retail services, transportation, and housing. For the evacuees who didn’t return, their employment to population ratio was more than half of the group of people who returned [14]. However, many financial aids were dispersed to them. Direct disaster relief amounted to about $100 billion [34]. Disaster Unemployment Assistance provided unemployment benefits for a period of up to 26 weeks to evacuees who were not covered by other unemployment insurance. It is estimated that charitable donations related to Hurricane Katrina were up to $4.25 billion [35]. All the disaster-related financial aid helped to maintain the evacuees’ purchasing power and as a result, increased the demand for locally produced goods and services.

The rising demand for local products can increase firm’s demand for inputs, which include their demand for labor inputs. And in fact, because of the additional demand from the non-workforce population and the postponed decision for new job search due to disaster assistance for the workforce population, the magnitude of the demand shock could be larger compared to the labor supply shock. However, for the industries whose products are more export-oriented, they would not be affected by local or regional demand shocks. This asymmetric changes in demand require a closer look at the industry attributes, which could generate different outcomes of wage rate in corresponding labor markets. To be more specific, for localized industries, the rise in population can increase firms’ demand for labor, which can offset the impact on wage rates from the labor supply shock; while for export-oriented industries, only the supply of labor is impacted due to these expected migrations.

In summary, the expectation with respect to the impact on local labor markets due to Katrina-induced migration can be illustrated in Fig 1 below:

**Fig 1:**
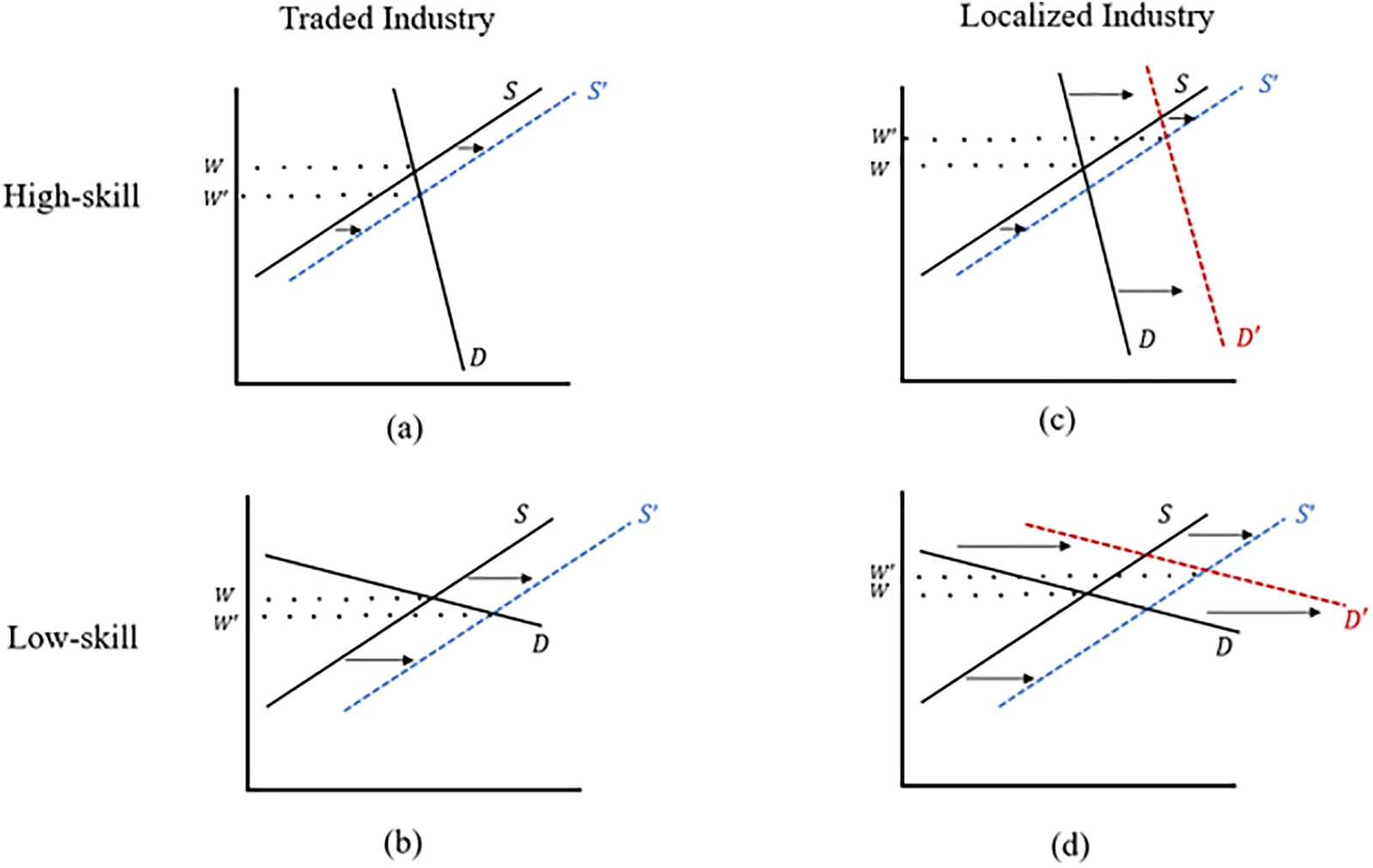
Expected impact from Katrina evacuees.

Panel (a) and (b) in Fig 1 illustrate the impact on traded industries by workers’ skill sets. Given that most of the Katrina evacuees were young and less-educated, a larger supply shock would happen in the low-skilled labor markets. However, as illustrated in panel (a) and (b), the labor demand curve is flatter for the low-skilled workers, in other words, the labor demand on the low-skilled workers is more elastic and the margins for wage change is smaller. As a result, the fall on wage rates could be smaller in the low-skilled traded industry, even though the shift of the supply curve is larger.

Panel (c) and (d) in Fig 1 illustrate the impact on localized industries by workers’ skill sets. Different from the traded industries, there is an additional demand shift in localized industries. The implication of both a demand and supply outward shift is that the impact on wages is uncertain. However, it is more likely to expect a rise in the wage rate for the high skilled workers since the labor supply shock by Katrina evacuees is smaller.

To summarize, following hypotheses are listed for empirical examination:

H1: The influx of Katrina evacuees negatively affects destination labor markets and exerts downward pressure on local wages.

H2a: Based on skill similarity, the negative wage impact of the evacuee influx is predominantly concentrated within the low-skilled labor market.

H2b: The labor market impact varies across sectors; in localized (non-traded) industries, the concurrent increase in consumer demand generated by evacuees partially offsets the negative supply-side shock.

H2c: The wage shock varies depending on the intensity of the evacuee inflow.

H2d: The wage impact on low-skilled native workers is constrained by a high elasticity of labor demand.

## 3. Data and methodology

### Data

In this study, we combine the Decennial Census data in 2000 and the American Community Survey (ACS) data from 2005 to 2008 to examine the effect of in-migration to the local economies due to Hurricane Katrina. Traditionally the Census collected data every 10 years from 1790 to 2000. In 2005, the Census began gathering data in a new way—the American Community Survey every year. Essentially the Decennial Census data and the ACS data are the same sources.

Since the American Community Survey was asking questions related to people’s situation in the past 12 months, for the vast majority of households surveyed in the 2005 ACS wave, the reference period covers months prior to August 2005. The labor market outcomes recorded in the 2005 data wave therefore captures pre-disaster conditions. It is only in the 2006 wave that the survey’s 12-month reference window fully captures the post-displacement labor supply shock. For this reason, we define 2006 as the initial post-treatment year.

To be consistent with the geographic areas used in the Census microdata, the unit of analysis used in this paper is called PUMA—the public used microdata area. PUMA is used by the US Census for statistical and demographic information. PUMA regions are better than counties in representing a local labor market due to its population lower bound being larger than 100,000, while the population level for counties in the U.S. ranges can be as low as a couple hundred.

The next question is to identify the evacuees from the survey data. In the Census survey, people were asked for their location of residence 1 year ago. In Xiao and Nilawar [36], a list of eight heavily damaged counties/parishes in Louisiana and Mississippi was offered, with an average of 46.9 percent of the occupied housing under major or severe damages. We then map these eight counties/parishes to the PUMA regions used in the Census data based on the population-weighted crosswalk from the Missouri Census Data Center. A total of six PUMAs were identified as Hurricane Katrina damaged areas. The crosswalk of the eight counties/parishes to the six areas is listed in S1 File Table A.1 with the population weight.

For each PUMA, we aggregate the number of migrants from the above six PUMAs to get the total number of migrants received due to Hurricane Katrina. Among a little over 2000 PUMA regions, there are about 550 PUMAs that have people migrated from the Katrina damaged area. To measure the intensity of this unexpected migration flows, we construct the ${Evacuee}_{i}^{\text{2}\text{0}\text{0}\text{5}}$ variable below:


$$\begin{array}{c} {Evacuee}_{i}^{\text{2}\text{0}\text{0}\text{5}}=\frac{No.ofevacueesfromKatrinaregiontoPUMAiin\text{2}\text{0}\text{0}\text{5}}{TotalpopulationinPUMAiin\text{2}\text{0}\text{0}\text{5}}-averagetrend_{i} \end{array}$$ (1)

$$\begin{array}{c} averagetrend_{i}=average_{year\text{1}\text{9}\text{9}\text{5}to\text{2}\text{0}\text{0}\text{0}}(\frac{No.ofmigrantsfromKatrinaregionto\;PUMAi}{TotalpopulationinPUMAi}) \end{array}$$ (2)

During normal years, there would be migrants from the six PUMAs to other places, and this normal trend was considered to construct the excessive flow due to Hurricane Katrina. The five-year average from the year 1995–2000 ($averagetrend_{i}$ in Equation (2)) represents the normal migration flow had Katrina not happened in 2005. Table 1 below summarizes the geographic distribution of Katrina evacuees, highlighting the six principal host states with the largest evacuee inflows along with the number of PUMAs experiencing strictly positive evacuee flows.

**Table 1:** Destination distribution of Katrina evacuee (top states by inflow).

Rank	State Name	Number of PUMAs Received Inflow
1	Texas	153
2	Louisiana	26
3	Mississippi	21
4	Florida	127
5	Georgia	63
6	Alabama	30

Source: Authors’ calculation.

The influx is heavily concentrated within the coastal states along the Gulf of Mexico (Listed in Table 1), or adjacent inland states such as Oklahoma. Statistically, descriptive analysis of variable ${Evacuee}_{i}^{\text{2}\text{0}\text{0}\text{5}}$ exhibits a highly right-skewed distribution (Skewness = 5.43) with an extremely heavy tail (Kurtosis = 39.61). Fig 2 Panel (a) below illustrates this extreme spatial heterogeneity by mapping PUMA-level treatment intensity variable ${Evacuee}_{i}^{\text{2}\text{0}\text{0}\text{5}}$.

**Fig 2:**
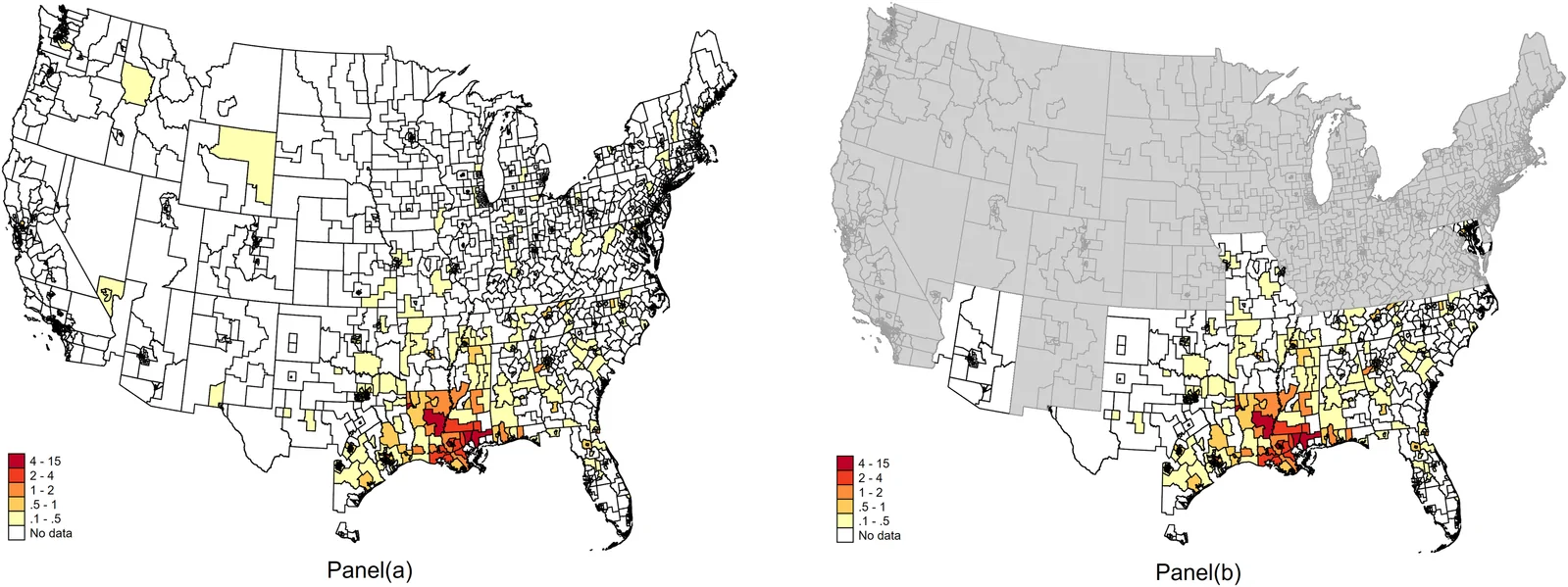
Inflow by PUMA regions (Unit: Percent of population). Notes: Map created by the authors using Stata software. Geographic PUMA boundary shapefiles are sourced from U.S. Census Bureau TIGER/Line files, which are in the public domain. Alaska, Puerto Rico and Hawaii are excluded.

The statistical and visual profile of variable ${Evacuee}_{i}^{\text{2}\text{0}\text{0}\text{5}}$ indicate that while the aggregate volume of the influx represents a historic and unprecedented volume, the treatment intensity is highly unequal and highly concentrated, leaving the vast majority of distant or insulated PUMAs unexposed (shaded in white in Fig 2 Panel (a)). Forcing thousands of these unexposed or close-to-zero-influx geographic units into the estimation introduces certain level of measurement noise, attenuating the magnitude of the estimated treatment coefficients toward zero and obscuring the true magnitude of the localized wage impact. To ensure methodological transparency regarding this transition, we report the benchmark regression using the full US sample in S1 File. Table A.2 (and discussed in Section 4).

As a result, regions with insufficient treatment intensity are excluded. Specifically, a state is excluded from our final estimation sample if: (i) it contains fewer than three PUMAs experiencing a strictly positive excess inflow, or (ii) its constituent PUMAs register an evacuee-to-population ratio below the 75th percentile of variable ${Evacuee}_{i}^{\text{2}\text{0}\text{0}\text{5}}$. These leaves 14 states, visually highlighted against the grayed-out background in Fig 2, panel (b).

Besides, right after Hurricane Katrina, Hurricane Rita hit the gulf coast again on September 15^th^, which was the fourth-most intense Atlantic hurricane ever recorded. Econometrically, retaining these units would confound the positive labor supply shock induced by Katrina evacuees with the negative local productivity shock induced by Rita’s physical damages. Hence, we exclude the regions that were heavily impacted by Hurricane Rita according to the report from the U.S. Department of Housing and Urban Development’s Office of Policy Development and Research. Specifically, to identify regions hit by Hurricane Rita, we measure physical damage using the proportion of heavily damaged properties. Given the right-skewed distribution of this variable, we exclude regions in the top quartile of physical damage—those exceeding the 75th percentile—to ensure our baseline estimates are unconfounded by severe local capital destruction.

All the above yields a final balanced panel comprising 672 PUMAs across 14 distinct states, containing 8 coastal states and 6 inland neighbor states. The survey data also provides each worker’s NAICS (North American Industry Classification System) code industry of work and his/her occupation, together with total wage income and total working hours. We then group people into the traded industry and localized industries based on the work of Delgado *et al.* [37]. They used the idea of geographic specialization and concentration of production activities in the U.S. to identify traded industries. To be more specific, for each industry, they examined their level of geographic concentration using the employment distribution and the Location Quotient (LQ), based on the regional employment data from the County Business Pattern. Three criteria was defined to distinguish the traded industries from the local industries that included: i) for an industry, its number of employees is less than 10 in more than 50 percent of the geographic regions, in other words, if that industry has employment concentrated in only a few regions; ii) the top 10 percent largest regions in that industry had the LQ being larger than 0.25; iii) the difference between the 90th percentile and 50th percentile LQ in that industry is larger than 1.5. We take their 6-digit NAICS industries that are traded or local and match them with the NAICS code in the Census survey microdata. Table A.3 and Table A.4 in the S1 File provided the list of local and traded industries used in the dataset.

Using the information on the total wage income and total working hours, we calculate worker’s hourly wage rate in each PUMA. Observations whose wage rate is below the minimum wage in the year 2000 or above the maximum confidentiality level are excluded. The background analysis for Hurricane Katrina suggests that the magnitude of the labor supply shock is larger for the low-skilled workers. To test this hypothesis, we classify people into low-skilled and high-skilled workers based on their occupations. Following the methodological framework in De Silva et al. [17], wage rates are utilized as a standard to distinguish skill levels and productivity. To eliminate potential endogeneity or feedback loops, we construct this measure using a static, pre-treatment national baseline. Specifically, we calculate the national average wage for each occupation using the 2000 ACS sample, which predates Hurricane Katrina and is entirely orthogonal to post-disaster localized shocks. Occupations in which the majority of the national workforce’s wage rate in 2000 was below the national average wage are defined as low-skilled occupations, and the remaining occupations are classified as high-skilled, and this classification is held strictly constant across all years and destination PUMAs in our panel. Table 2 below presents the distribution of Katrina evacuees in these four different markets.

**Table 2:** Distribution of the evacuees by industries and skills.

	Localized Industry	Traded Industry
Low-Skilled	48%	20%
High-Skilled	16%	16%

After defining each worker’s industry and skill set, we obtain weighted wage rate by PUMA region under the four types of markets that are traded-low-skill, traded-high-skill, local-low-skill, and local high-skill.

There were still chances for a second or a third migration for these evacuees before they finally settled down from the impact of Hurricanes. And this additional migration may be associated with potential job opportunities or other economic factors that can determine the supply of labor as well as the wage rate in local markets. Hence, certain factors that can impact both migration and labor supply were considered:

Eyer *et al.* [38] found that Katrina migrants tend to move to areas with higher average pay. Human capital in a region can help decide the level of average pay in the following sense: All else equal, a region with a larger percentage of college and above graduates can have higher average pay. We consider the PUMA level education attainment to proxy for the average payment level in that PUMA region.

According to Ehrenberg and Smith [39], a factor that impacts immigrants’ decision on whether to enter into the job market or what type of job market is their language proficiency. The level of language proficiency in the local region can also influence people’s migration decisions. After Hurricane Mitch, Kugler and Yuksel [7] found that residents migrated out since the evacuees from Latin American arrived. Similarly, the English proficiency level can be used to proxy this kind of local features that guide people’s migration behavior. The Census survey asked about people’s English proficiency level and we calculate the percent of adults who didn’t speak English or speak little English for every PUMA to represent language proficiency in local labor markets.

The summary statistics of the major variables used in this paper is in Table 3, which is structured into two distinct panels to accurately reflect the underlying temporal and econometric variation of our data. Panel A reports the time-varying outcome variables and a mediating variable; Panel B focuses on time-invariant and baseline control variables. Our primary explanatory measures belong to the latter category, functioning as the foundational components of our Difference-in-Differences (DID) framework, which will be discussed in detail in the following subsection.

**Table 3:** Summary statistics.

Variable	Observations	Mean	Std. Dev.	Min	Max
**Panel A: Time-Varying Variables (2000, 2005–2008)**					
Wage Rates (Low-Skill Localized Industry)	3,360	13.30	1.750	8.749	21.13
Wage Rates (High-Skill Localized Industry)	3,360	24.67	4.134	15.15	43.33
Wage Rates (Low-Skill Traded Industry)	3,360	16.58	2.895	10.55	30.28
Wage Rates (High-Skill Traded Industry)	3,360	28.08	5.803	13.49	49.20
Number of Firm Establishment	3,360	9931	3500	3003	32275
**Panel B: Baseline Characteristics (Time Invariant)**					
Area Experienced Evacuee Inflow in 2005 (binary)	672	0.52	0.500	0	1
Evacuee Inflow/Population	672	0.24%	0.006	0	7.12%
Percent with no more than High school Diploma (year 2000)	672	46.5%	0.094	0.145	64.9%
Percent whose English is poor (year 2000)	672	3.25%	0.046	0.14%	35.4%

### Methodology

To determine the impact of Hurricane-induced displacement on the regional labor market, we estimate the following baseline specification:


$$\begin{array}{c} Y_{it}=\alpha \left({Treat}_{i}^{\text{2}\text{0}\text{0}\text{5}}\times Post_{t}\right)+X_{it}^{T}\beta +\delta_{i}+Year_{t}+\epsilon_{it} \end{array}$$ (3)

where i indexes PUMAs and t indexes time-period for 2000, 2005–2008.$Y_{it}$ is the outcome variable that is the wage rate for the four types of industries. ${Treat}_{i}^{\text{2}\text{0}\text{0}\text{5}}$ is a dummy variable that equals to 1 for area experienced excessive evacuees. $Post_{t}$ is a dummy variable that equals 1 for years after 2005. We include both PUMA fixed effects $\delta_{i}$ and time-period fixed effects $Year_{t}$, in our baseline specification. PUMA fixed effects account for all time-invariant characteristics that vary across counties. Time-period fixed effects account for any secular changes over time that affect all PUMAs equally. The coefficient of interest in the equation is α. It measures the change in wage rates with in-migration due to Hurricane Katrina. As discussed in the theoretical section above, α should be negative for the export-oriented industry due to the rise in labor supply, but the sign of α is uncertain for the localized industries with a higher chance of being positive for the high-skilled workers.

An important empirical validation of Difference in Difference is the parallel trend before treatment. To determine whether it is the case we examine how the relationship between Katrina-induced migration in 2005 and our outcome variables evolve over time by estimating the following fully flexible specification:


$$\begin{array}{c} Y_{it}=\Gamma_{t}\left({Treat}_{i}^{\text{2}\text{0}\text{0}\text{5}}\times Year_{t}\right)+X_{it}^{T}\beta +\delta_{i}+Year_{t}+\epsilon_{it} \end{array}$$ (4)

where all variables are defined as in Equation (3) with one exception that in Equation (4), rather than interacting the treatment variable with a post Hurricane dummy variable, we interact it with the full set of time-periods fixed effects. And the resulting series of the estimated coefficients $\Gamma_{t}$ represent the relationship between displacement and outcome variables in each time period. If, for example, Hurricane-induced migration decreased wages then we would expect the estimated coefficients to be insignificant in the pre-treatment period. In this way, the fully flexible specification in Equation (4) allows for a closer examination of the patterns in the data to determine whether there is any trend difference between the treatment and control groups before Hurricane Katrina.

## 4. Results

**Baseline Estimates:** Table 4 reports the baseline regression results focusing on the wage rate of the low-skill localized industry, which is the only labor market segment among the four examined to be significantly affected by the shock. And this finding is partially consistent with H1 and H2a in Section 2.

**Table 4:** Baseline regression results.

VARIABLES	(1)	(2)
	Wage Rate	Wage Rate
	(Low-skill local)	(Low-skill local)
${Treat}_{i}\times Post_{t}$	−0.160***	−0.183***
	(0.049)	(0.049)
Observations	3,360	3,360
Number of Puma	672	672
Adjusted R-squared	0.492	0.503
Controls	NO	YES
PUMA FE	YES	YES
Cluster SE	YES	YES
Time FE	YES	YES

Standard errors in parentheses.

*** p < 0.01, ** p < 0.05, * p < 0.1.

Column (1) in Table 4 presents the Difference in Difference estimates excluding any baseline covariates. The estimated coefficient on the interaction term is −0.160, which is statistically significant at the 1% level. This suggests that on average, PUMA areas with Katrina evacuees lead to a 0.16 dollar fall in the hourly wage rates. In Column (2), we include the pre-Katrina baseline controls (the local proportion of high school graduates and non-English speakers in 2000, before the Katrina event) interacted with year dummies. The coefficient remains significant at 1% level, but the magnitude of the wage fall becomes larger. From an economic perspective, these results indicate that the labor supply shock induced by the evacuee influx exerted a robust downward pressure on the wage rates within the low-skill localized industry. While H1 predicts a general wage decline, our results clarify that there is no uniform downward shift across the entire wage distribution, but only valid at the low-skilled localized industry. H2a put skill as an important factor for the supply pressure, but still lacks ability to explain the asystemtric impact between local and traded industry.

Importantly, given that the average hourly wage for low-skilled workers in these localized industries is 13.3 dollars, this 0.16-dollar decline represents a relatively modest wage reduction of approximately 1.2%. This limited impact supports Hypothesis 2d, suggesting that the wage decline is heavily constrained by a high elasticity of labor demand.

A core identifying assumption of the Difference-in-Differences framework is the parallel trends assumption, which requires that the trajectories of the outcome variables would be identical across treatment and control groups before the hurricane shock. To evaluate the validity of this assumption, we implement an event-study specification across all four distinct labor markets. Given that our pre-treatment window spans two distinct periods, we omit the initial year (2000) as the reference category to avoid multicollinearity. The dynamic specification estimates the treatment intensity interaction for the remaining pre-treatment period (2005) immediately preceding the influx.

Table 5 reports the pre-trend estimation results for the four labor market categories: low-skill localized (Market1), low-skill tradable (Market2), high-skill localized (Market3), and high-skill tradable (Market4). The empirical results show that the coefficient for the pre-treatment interaction term is statistically indistinguishable from zero across all four markets.

**Table 5:** Pre-trend analysis in four labor markets.

VARIABLES	(1)	(2)	(3)	(4)
	Market1	Market2	Market3	Market4
${Treat}_{i}\times {Year}_{\text{0}\text{5}}$	−0.023	0.035	−0.095	0.395
	(0.076)	(0.150)	(0.190)	(0.299)
Observations	3,360	3,360	3,360	3,360
Number of Puma	672	672	672	672
Adjusted R-squared	0.492	0.248	0.426	0.266
PUMA FE	YES	YES	YES	YES
Cluster SE	YES	YES	YES	YES

Standard errors in parentheses.

*** p < 0.01, ** p < 0.05, * p < 0.1.

Fig 3 plots the estimated coefficients if Equation (4) along with their corresponding 95% confidence intervals for low-skill localized industry wages. As illustrated in Fig 3 below, the pre-treatment coefficients are statistically indistinguishable from zero. Following the evacuee influx, the post-treatment coefficients across all subsequent periods are statistically significant and negative. This dynamic pattern confirms that the labor supply shock exerted a persistent negative impact on wages in the low-skill localized industry.

**Fig 3:**
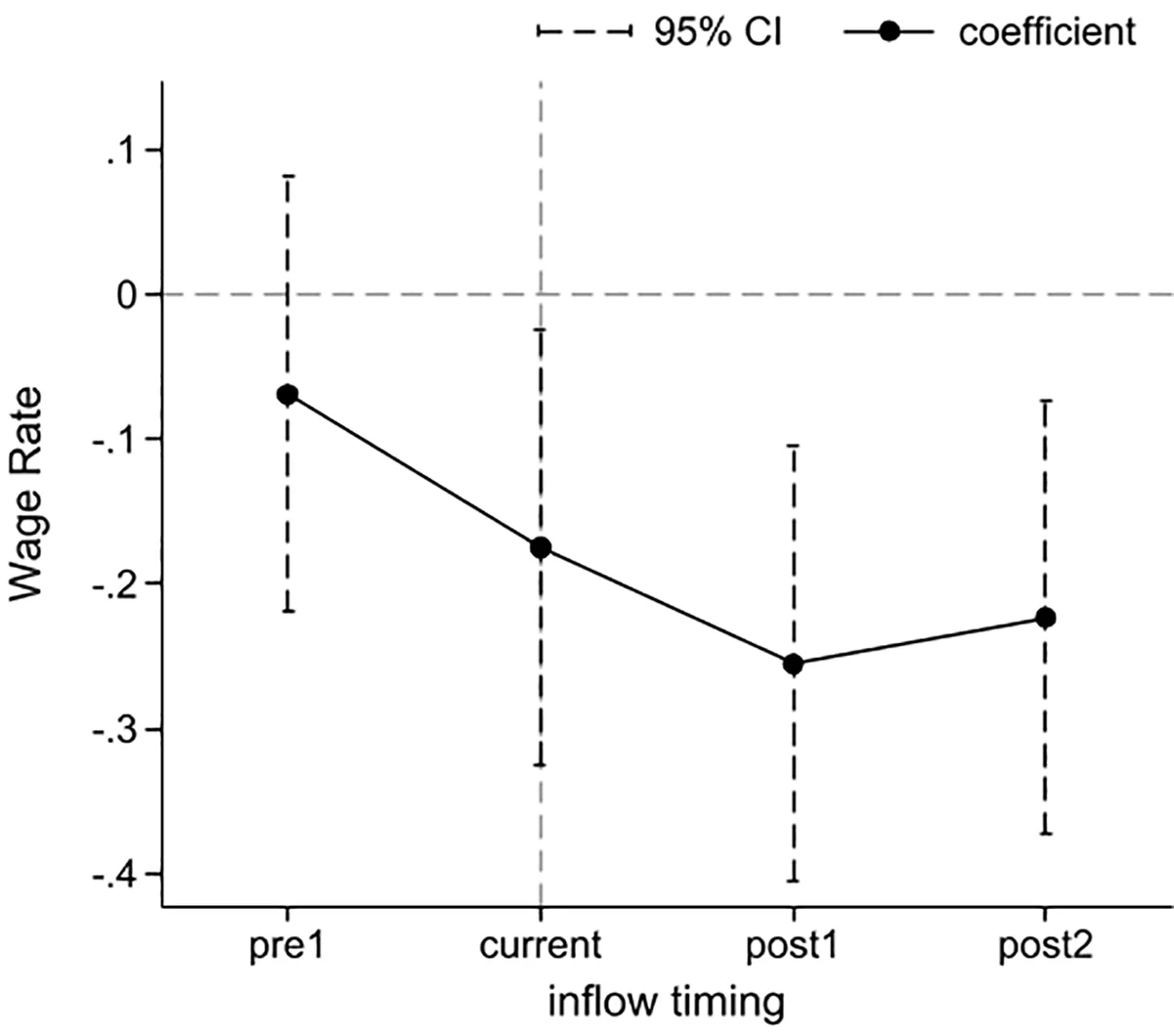
Parallel trend for low-skill localized labor market (Source: Authors’ calculation).

**Intensity check:** To further scrutinize the potential non-linear and threshold effects of the evacuee influx, we partition the treatment intensity into four distinct regimes based on the empirical distribution of ${\text{Evacuee}}_{\text{i}}$. The distribution exhibits a substantial right-skewness and a heavy-tailed kurtosis (Fig 4), highlighted by the fact that the median influx rate (0.20%) is drastically lower than the sample maximum (7.12%).

**Fig 4:**
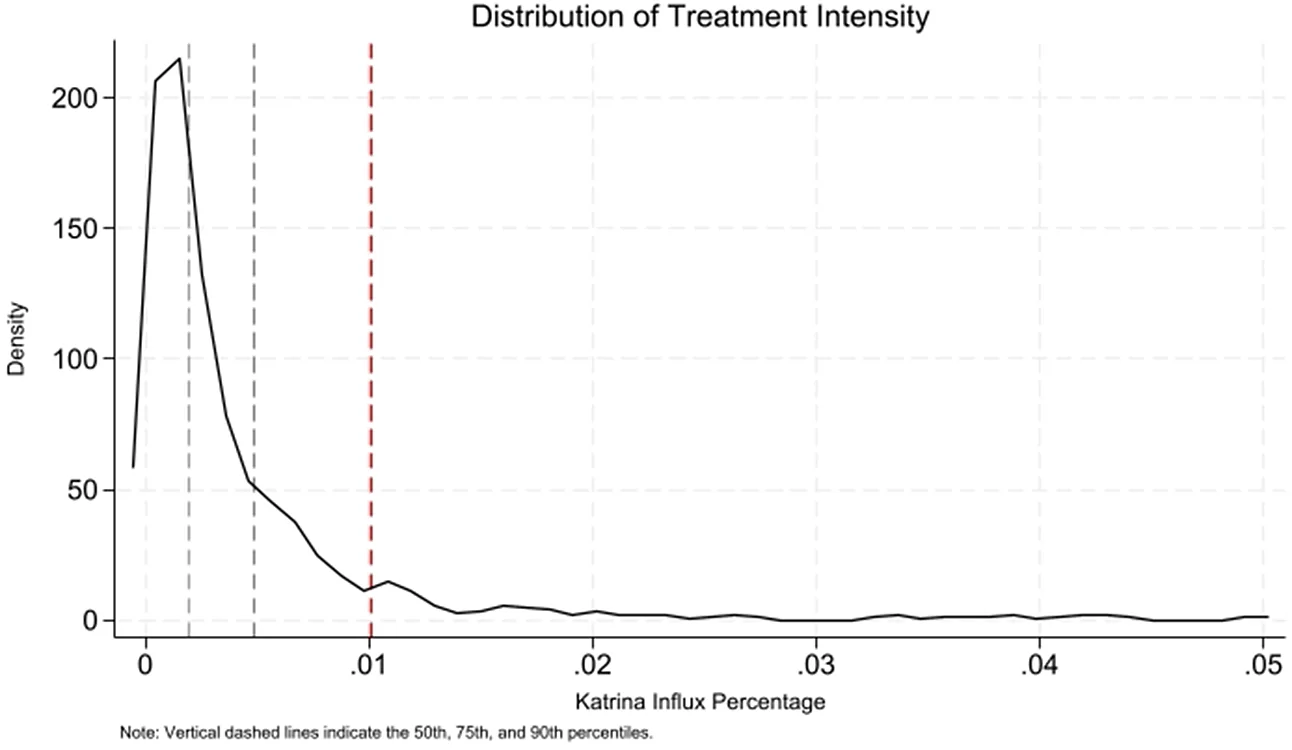
Distribution of evacuee to population ratio (Source: Authors’ calculation).

Under such a distributional profile, standard linear or symmetric quantile specifications would fail to isolate the disproportionate leverage exerted by extreme influx destinations. Therefore, we deliberately segregate the active sample into group1 (0–50th percentile), group2 (50th–75th percentile), and group3 (75th–100th percentile). Table 6 reports the regression results estimating the differential impacts of these intensity groups on the wage rate of the low-skill localized industry.

**Table 6:** Intensity analysis.

VARIABLES	(1)
	Wage Rate (Low-skill local)
${Group\_\text{1}}_{i}\times Post_{t}$	−0.152**
	(0.060)
${Group\_\text{2}}_{i}\times Post_{t}$	−0.156**
	(0.077)
${Group\_\text{3}}_{i}\times Post_{t}$	−0.273***
	(0.077)
Observations	3,360
Number of Puma	672
R-squared	0.604
PUMA FE	YES
Cluster SE	YES

Standard errors in parentheses.

*** p < 0.01, ** p < 0.05, * p < 0.1.

The empirical findings reveal a distinct non-linear pattern in the wage effect relative to evacuee concentration. In Table 6, for PUMAs in Group 1 (the 0−50th percentile range), the estimated coefficient on the interaction term is −0.152, which is statistically significant at the 5% level. For Group 2 (the 50−75th percentile range), the wage reduction remains stable. Crucially, the negative wage effect intensifies substantially in the upper tail of the treatment distribution. For PUMAs facing the highest treatment intensity in Group 3(the 75−100th percentile range), the coefficient drops sharply to −0.273, achieving statistical significance at the 1% level. This finding is consistent with hypothesis H4a.

From an economic perspective, these graded estimates indicate that while low-to-moderate influxes trigger a baseline contraction in localized wages, extreme concentrations of evacuees compound the local labor supply shock. This disproportionate wage depression in Group 3 confirms that local low-skill labor markets experience severe, non-linear effects when the volume of exogenous labor inflows reaches the highest percentiles.

**More robustness check:** To maintain complete methodological transparency regarding our sample construction process, Table A.2 in S1 File reports our baseline specifications estimated on the unrefined universe of U.S. PUMAs. Under this full-sample specification, the estimated treatment coefficient for the low-skill localized sector remains negative and highly statistically significant at 1% level (β=−0.124). However, the magnitude is substantially attenuated compared to our primary 14-state estimation sample (β=−0.183). This attenuation confirms that including thousands of distant, completely unexposed geographic units introduces measurement noise that washes out the localized wage response.

Crucially, the estimated effects for the remaining labor market sectors (high-skill localized, high-skill traded, and low-skill traded) remain statistically insignificant across the national sample, preserving the sectoral specificity observed in our primary estimates.

Besides, when restricting the pre-treatment window exclusively to 2005 (Table A.5 in S1 File), the estimated wage reduction for the low-skill localized sector remains negative and statistically significant at the 5% level (β=−0.148). The slight attenuation in coefficient magnitude reflects the removal of multi-year accumulated trends, confirming that our core conclusions are robust to alternative pre-treatment window definitions. In alignment with our baseline findings, treatment estimates across all other labor market sub-sectors remain consistently insignificant, confirming that our core results are neither artifacts of long-term trend differences nor driven by broad regional wage spillovers.

## 5. Mechanism analysis

**Demand shift mediator:** In De Silva *et al.* [17], firm-level total sales data is used to control for the potential demand-side impact that was brought by these evacuees. Similar information is not available publicly. But there is another set of information from the non-employer statistics (NES), which recorded the NACIS industry level total sales data for the businesses that have no paid employers but are subject to federal income tax. Even though only accounting for about 4 percent of all sales nationally, the majority of all business establishments in the United States are non-employers. This information can also reflect the demand hikes due to the inflow of the evacuees. For example, home buyers or rental agents can belong to the category of non-employer businesses and receive a rise in demand from the evacuees to settle down in the new area. The NES data is provided at the county level, we match it by population weighting matrix to PUMA level, which is the same method used earlier in obtaining the Katrina damaged PUMA regions.

However, prior research suggests that an immigration shock can significantly depress output prices in the affected sectors. In response to a sudden influx of low‑skill immigrants, firms tend to shift toward lower value‑added goods and services, in order to accommodate the skill composition of the new workforce and maintain market competitiveness [40]. Therefore, the number of firms is used as our core mediator. Equations (5) to (6) below are used to account for this demand-side impact:


$$\begin{array}{c} {Estab}_{it}=\theta_{\text{1}}\left({Treat}_{i}\times {Post}_{t}\right)+X_{it}^{T}\Theta +\mu_{i}+{Year}_{t}+\varepsilon_{it} \end{array}$$ (5)

$$\begin{array}{c} Y_{it}=\gamma_{\text{1}}\left({Treat}_{i}\times Post_{t}\right)+\gamma_{\text{2}}{Estab}_{it}+X_{it}^{T}\Upsilon +\delta_{i}+Year_{t}+\epsilon_{it} \end{array}$$ (6)

where ${Estab}_{it}$ is the number of firm establishments for PUMA i in year t.

Table 7 reports the empirical estimates of Equations (5) and (6). Column (1) replicates our baseline treatment effect on localized wages within this sample, Column (2) evaluates the first stage of the mediation path, assessing whether the evacuee shock stimulated firm creation. The coefficient on DID variable is positive and statistically significant at the 10% level, and the positive sign is consistent with our hypothesis H3a. Column (3) estimates the complete specification. The direct effect of the mediator is positive and statistically significant at the 5% level, indicating that firm expansion is associated with higher local labor demand. Once we account for this mediator pathway, the direct coefficient of the evacuee shock on wages shifts slightly to −0.187.

**Table 7:** Mediation effect results.

VARIABLES	(1)	(2)	(3)
	Wage	Firm Establishment	Wage
${Estab}_{it}$			0.040**
			(0.019)
${Treat}_{i}\times Post_{t}$	−0.183***	0.098*	−0.187***
	(0.049)	(0.050)	(0.049)
Observations	3,360	3,360	3,360
Number of puma1	672	672	672
Adjusted R-squared	0.503	0.751	0.503
Controls	YES	YES	YES
PUMA FE	YES	YES	YES
Cluster SE	YES	YES	YES
Time FE	YES	YES	YES

Standard errors in parentheses.

*** p < 0.01, ** p < 0.05, * p < 0.1.

Results in Table 7 indicates that the negative wage pressure of the labor supply shock is marginally offset by the demand-side response of firm creation, illustrating the structural adjustment process toward a new local labor market equilibrium. However, the coefficient on firm establishments is only significant at the 5% level, and its absolute magnitude remains economically small. Therefore, the empirical evidence supports the likelihood of a demand-side adjustment channel, but indicates that its countervailing effect was ultimately marginal, compared to the dominant negative pressure exerted by the supply shock.

**Flow across labor markets:** A key conceptual and methodological assumption of our empirical design is that the designated labor markets (defined by the intersection of skill levels and trade structures) operate with a sufficient degree of independence. However, if skills are highly transferable across sectors, a localized wage decline in one market could induce native workers to relocate to other industries within the same region. Such cross-market labor flows would violate the partial equilibrium assumption and potentially bias our baseline estimates.

To test the validity of this independence assumption and empirically evaluate whether native workers migrated across sectors in response to the evacuee-induced wage shock, we construct measures of the sector-specific employment shares of native workers (defined as individuals who did not migrate within the past 12 months).

Specifically, we focus on the potential labor flows between the localized and tradable sectors within the low-skill segment, as this group is the most exposed to the labor supply shock. We estimate the impact of the evacuee influx on these employment shares using our Difference-in-Differences specification. Table 8 reports these results.

**Table 8:** Labor flow in low-skilled market.

VARIABLES	(1)	(2)	(3)
	Ln(ratio1) Localized Industry	Ln(ratio2/ratio1) Traded vs Local	Ln(ratio2) Traded Industry
${Treat}_{i}\times Post_{t}$	0.001	0.029**	0.034***
	(0.005)	(0.013)	(0.011)
Observations	2,688	2,688	2,688
Number of Puma	672	672	672
Controls	YES	YES	YES
PUMA FE	YES	YES	YES
Cluster SE	YES	YES	YES
Time FE	YES	YES	YES

Note: Year 2000 is excluded due to unavailable information.

Standard errors in parentheses.

*** p < 0.01, ** p < 0.05, * p < 0.1.

Column (1) in Table 8 reports the impact of the evacuee shock on the log share of native workers employed in the low-skill localized sector (ratio1). The estimated coefficient is statistically insignificant, indicating the shock did not trigger a significant absolute exit of native workers from the localized industry. In Column (2), we examine the log ratio of native employment in the localized sector relative to the tradable sector (ratio2/ratio1) to capture relative structural shifts. This positive and significant estimate indicates that the share of native low-skill employment in the localized sector actually increased relative to the tradable sector. To ensure that this finding is not a statistical artifact of the log-ratio transformation or the linear fixed-effects specification, Column (3) models the log share of native workers in the tradable sector (ratio2) using a Generalized Estimating Equations (GEE) framework. The coefficient is positive and statistically significant at the 1% level.

From an economic perspective, these results provide no evidence of native labor flight from the localized sector to the tradable sector. If native workers were fleeing the wage-depressed localized sector, we would expect a negative coefficient in Column (1) and a positive coefficient in Column (3) representing an increase in the tradable share relative to the localized share. Instead, the empirical patterns indicate that native low-skill workers remained in the localized sector, while employment in the tradable sector grew. This lack of systematic displacement of native labor across sectors supports our empirical methodology.

To ensure the robustness of our sectoral independence assumption across the entire labor supply distribution, we replicate the sectoral mobility analysis for the high-skill labor market. In the high-skill localized industry, the estimated treatment coefficient is statistically indistinguishable from zero, in the high-skill tradable sector, the estimated coefficient is slightly negative and marginally significant at the 10% level. This marginal contraction in the high-skill tradable employment share likely reflects general equilibrium constraints or localized capital-labor adjustments following the shock. Crucially, the absence of a corresponding positive labor flow into the high-skill localized sector rules out any systemic, wage-driven occupational sorting or arbitrage across these high-skill markets in the short run.

**Joint determination of sector wages:** As another robustness check, a remaining empirical concern is that wages across different labor markets may be jointly determined, even though labor market flows are not clearly observed. To address this possibility of general equilibrium linkages and formally test the independence of the wage-setting processes, we specify a simultaneous system of equations and estimate it using a Seemingly Unrelated Regressions (SUR) framework. Table 9 reports the parameter estimates and the corresponding cross-equation independence test.

**Table 9:** General equilibrium simultaneous system estimations results.

VARIABLES	(1)	(2)
	Wage Rate (Low-skill local)	Wage Rate (Low-skill Traded)
${Treat}_{i}\times Post_{t}$	−0.160***	0.049
	(0.044)	(0.086)
Observations	3,360	3,360
Adjusted R-squared	0.594	0.400
Controls	NO	NO
PUMA FE	YES	YES
Time FE	YES	YES
Breusch-Pagan Test of Independence p-value	0.1452	

Standard errors in parentheses.

*** p < 0.01, ** p < 0.05, * p < 0.1.

The system estimation results confirm the robustness of our baseline findings. In Column (1), the estimated coefficient on the demeaned evacuee shock for the low-skill localized sector is statistically significant at the 1% level, and the magnitude is similar to the initial regression results in Table 4 above. Column (2) reveals that the evacuee shock has a positive but statistically insignificant effect on wages in the low-skill tradable sector, which also remains unchanged.

To evaluate the validity of the structural independence between these two labor markets, we perform a Breusch-Pagan test of independence on the residual covariance matrix. P-value of 0.1452 making us fail to reject the null hypothesis that the contemporaneous covariance between the residuals of the two equations is zero.

Consequently, the simultaneous equation analysis provides no evidence of general equilibrium spillovers or wage co-determination across these sectors, validating our conceptualization and estimation of these labor markets as independent economic units.

## 6. Conclusion and discussion

This paper provides a systematic empirical evaluation of how a sudden labor supply shock—instigated by a large-scale evacuee influx—afflicts localized labor markets. Empirically, we leverage a Difference-in-Differences framework at the Public Use Microdata Area (PUMA) level, utilizing a five-year panel dataset spanning the period immediately preceding and following the Hurricane Katrina displacement shock. This localized panel design allows us to track high-frequency labor market adjustments within narrowly defined geographic boundaries.

Our baseline and threshold analyses reveal that the displacement shock consistently exerts downward pressure on low-skill localized industry wages, with an average impact to hourly wage rates by 0.18 dollars, this negative impact is also heavily characterized by non-linear effects. Specifically, the wage contraction remains relatively stable within the 75th percentiles of the treatment distribution, but intensifies sharply (from −0.152 to −0.273 dollars) above this threshold.

To explain this adjustment process, our structural decomposition of demand-side channels indicates that although the population influx stimulated product market demand and local business creation, which in turn associated positively with wages, this countervailing channel is economically trivial. Consequently, short-run firm creation and demand-side scaling remain highly constrained and are ultimately insufficient to fully absorb the localized supply shock.

Methodologically, our causal identification relies on the structural independence of the designated labor markets, a premise that we validate through multi-dimensional empirical tests. The sectoral mobility analysis indicates that the shock did not trigger a systematic exit of native low-skill workers from the localized industry, suggesting that occupational barriers and task-specific skill requirements induce significant short-run labor market segmentation. Furthermore, a simultaneous equation system estimated via Seemingly Unrelated Regressions fails to reject the null hypothesis of residual orthogonality. This implies that once time-invariant geographic and temporal con-founders are controlled, there are no unobserved general equilibrium linkages or joint wage-setting dynamics across the localized and tradable sectors.

The non-linear negative shock on localized low-skill wages deepens our understanding of spatial adjustments following sudden demographic displacements, offering a crucial refinement to existing literature. While early assessments of the Hurricane Katrina migration, such as McIntosh [8], tend to report modest or statistically weak average labor market impacts in host cities like Houston, our threshold-specific estimations demonstrate that average effects conceal significant spatial heterogeneity. This empirical pattern suggests that host labor markets can absorb displaced workers relatively well up to a certain threshold, beyond which acute non-linear congestion effects manifest.

This duration-dependent wage pressure is further illustrated when contrasting our results with preventive, ultra-short-term evacuations. For instance, as documented by Siebeneck and Cova [41], in Hurricane Rita return-entry process, approximately 2.5 million evacuees evacuated the Texas coast but the vast majority returned to their origins within days to weeks. Because of this extremely brief displacement period, Rita evacuees result in no detectable long-run wage suppression in host cities like Houston or Austin.

Our second set of results shows a relative increase in the share of native workers within the low-skill traded sector. Studies show that low-skill supply shocks trigger alternative occupational sorting and defensive sectoral upgrading along the wage distribution (Dustmann et al. [42]). While conceptually plausible, this selective sorting of new entrants is unlikely to confound our main baseline identifying assumption for two reasons: first, our primary labor flow analysis tracks the established, non-migrant native population, purging the mechanical influence of highly mobile marginal entrants; second, our Seemingly Unrelated Regressions (SUR) baseline residuals exhibit complete contemporaneous orthogonality, confirming that sectoral wage-setting processes remain segmented in the short run. An alternative hypothesis is that the relative expansion of native employment in the tradable sector is driven by new labor market entrants. Future research utilizing individual-level longitudinal registry data could further trace the exact occupational trajectories of these new entrants to map the micro-foundations of this sectoral barrier.

Finally, our structural decomposition reveals that although the evacuee influx stimulated localized product demand and business creation, this demand-side buffer was economically modest and insufficient to offset the supply-driven wage depression. This result stands in contrast to the classic findings of Bodvarsson et al. [43] on the Mariel Boatlift, which argue that immigration-induced demand-creation can almost entirely neutralize supply-side wage pressure. We reconcile this divergence by highlighting the unique friction of disaster-induced displacements. Unlike steady, predictable immigration flows, sudden catastrophic shocks present extreme short-run adjustment frictions. As theorized by Lewis [44], capital-skill complementarity and technological adjustments within firms require substantial lead times and stable policy horizons. In the immediate aftermath of Hurricane Katrina, heightened economic uncertainty and capital adjustment frictions severely constrained the capacity of new and existing firms to scale up, explaining why the demand-side mitigation channel remained statistically detectable but economically marginal in the short run.

These empirical patterns offer highly specific, localized policy implications. First, the presence of severe non-linear congestion effects suggests that the geographic concentration of displaced populations, rather than the aggregate volume of the influx per se, is the primary driver of rapid wage depreciation. Policymakers managing sudden demographic shocks should prioritize geographic dispersion strategies over concentrated resettlement to mitigate localized wage shocks. Second, because the short-run capacity of local firm creation and demand-side buffers to absorb excess labor is statistically detectable but economically marginal, public intervention cannot rely on spontaneous private-sector adjustment to restore equilibrium. Targeted transition assistance, temporary wage subsidies, or retraining programs designated to lower occupational entry barriers between localized and tradable industries may be required to facilitate orderly labor reallocation and alleviate localized labor market distress.

## Supporting information

S1 File Additional tables. (DOCX)

S2 File Dataset. (RAR)
