# Supplementary material for: Disaster-induced migration and local labor markets: Heterogeneous effects by skill level and industry structure
Source: PLoS One. 2026 Oct 27;21(10):e0346313. doi: 10.1371/journal.pone.0346313 (PMC13712529; doi:10.1371/journal.pone.0346313)
Supplement: Supplementary file 1 [file pone.0346313.s001.docx]

Appendix

Table A.1 Crosswalk from Katrina Damaged County/Parish to Migration PUMA region

| County | State | County FIPS Code | Migration PUMA | Weight |
| --- | --- | --- | --- | --- |
| Jefferson | Louisiana | 22051 | 1900 | 1 |
| Orleans | Louisiana | 22071 | 1800 | 1 |
| Plaquemines | Louisiana | 22075 | 1900 | 1 |
| St Bernard | Louisiana | 22087 | 1900 | 1 |
| St Tammany | Louisiana | 22103 | 2000 | 0.927 |
| Hancock | Mississippi | 28045 | 2100 | 0.827 |
| Harrison | Mississippi | 28047 | 2200 | 1 |
| Jackson | Mississippi | 28059 | 2300 | 1 |

***Note:*** *The weight means that to what extent the migration PUMA region overlaps with the county/parish boundary.*

Table A.2 Sample Sensitivity Regression Results

|  | (1) | (2) | (3) | (4) |
| --- | --- | --- | --- | --- |
| VARIABLES | Market1 | Market2 | Market3 | Market4 |
| ${Treat}_{i}\times Post_{t}$ | -0.124*** | -0.100 | -0.106 | -0.015 |
|  | (0.034) | (0.062) | (0.086) | (0.116) |
|  |  |  |  |  |
| Observations | 10,225 | 10,225 | 10,225 | 10,225 |
| Number of Puma | 2,045 | 2,045 | 2,045 | 2,045 |
| Adjusted R-squared | 0.496 | 0.308 | 0.380 | 0.306 |
| Controls | YES | YES | YES | YES |
| PUMA FE | YES | YES | YES | YES |
| Cluster SE | YES | YES | YES | YES |
| Time FE | YES | YES | YES | YES |

***Notes:*** *Standard errors in parentheses *** p<0.01, ** p<0.05, * p<0.1*

*The four labor market categories are: low-skill localized (Market1), low-skill tradable (Market2), high-skill localized (Market3), and high-skill tradable (Market4).*

Table A.3 Localized Industries and the Corresponding NAICS

| Industry Name | NAICS |
| --- | --- |
| Natural gas distribution | 2212P |
| Sewage treatment facilities | 22132 |
| Construction | 23 |
| Retail bakeries | 311811 |
| Motor vehicles, parts and supplies | 4231 |
| Lumber and other construction materials | 4233 |
| Hardware, plumbing and heating equipment, and supplies | 4237 |
| Recyclable material | 42393 |
| Groceries and related products | 4244 |
| Automobile dealers | 4411 |
| Other motor vehicle dealers | 4412 |
| Auto parts, accessories, and tire stores | 4413 |
| Furniture and home furnishings stores | 442 |
| Household appliance stores | 443111 |
| Radio, tv, and computer stores | 4431M |
| Hardware stores | 44413 |
| Building material and supplies dealers | 4441Z |
| Lawn and garden equipment and supplies stores | 4442 |
| Grocery stores | 4451 |
| Specialty food stores | 4452 |
| Beer, wine, and liquor stores | 4453 |
| Pharmacies and drug stores | 44611 |
| Health and personal care, except drug, stores | 446Z |
| Gasoline stations | 447 |
| Shoe stores | 44821 |
| Jewelry, luggage, and leather goods stores | 4483 |
| Clothing and accessories, except shoe, stores | 448ZM |
| Sewing, needlework and piece goods stores | 45113 |
| Book stores and news dealers | 45121 |
| Music stores | 451M |
| Department stores | 45211 |
| Miscellaneous general merchandise stores | 4529 |
| Florists | 4531 |
| Office supplies and stationary stores | 45321 |
| Gift, novelty, and souvenir shops | 45322 |
| Used merchandise stores | 4533 |
| Miscellaneous stores | 4539 |
| Vending machine operators | 4542 |
| Fuel dealers | 45431 |
| Other direct selling establishments | 45439 |
| Truck transportation | 484 |
| Bus service and urban transit | 485M |

Table A.3 Localized Industries and the Corresponding NAICS continued…

| Industry Name | NAICS |
| --- | --- |
| Couriers and messengers | 492 |
| Sporting goods, camera, and hobby and toy stores | 4M |
| Not specified trade | 4MS |
| Newspaper publishers | 51111 |
| Wired telecommunications carriers | 5171 |
| Radio and television broadcasting and cable | 51M |
| Insurance carriers and related activities | 524 |
| Banking and related activities | 52M1 |
| Real estate | 531 |
| Automotive equipment rental and leasing | 5321 |
| Videotape and disk rental | 53223 |
| Other consumer goods rental | 532M |
| Legal services | 5411 |
| Accounting, tax preparation, bookkeeping and payroll services | 5412 |
| Veterinary services | 54194 |
| Employment services | 5613 |
| Business support services | 5614 |
| Investigation and security services | 5616 |
| Landscaping services | 56173 |
| Services to buildings and dwellings | 5617Z |
| Waste management and remediation services | 562 |
| Elementary and secondary schools | 6111 |
| Offices of physicians | 6211 |
| Offices of dentists | 6212 |
| Office of chiropractors | 62131 |
| Offices of optometrists | 62132 |
| Offices of other health practitioners | 6213ZM |
| Outpatient care centers | 6214 |
| Home health care services | 6216 |
| Other health care services | 621M |
| Hospitals | 622 |
| Nursing care facilities | 6231 |
| Residential care facilities, without nursing | 623M |
| Individual and family services | 6241 |
| Community food and housing, and emergency services | 6242 |
| Vocational rehabilitation services | 6243 |
| Child day care services | 6244 |
| Bowling centers | 71395 |
| Traveler accommodation | 7211 |
| Drinking places, alcohol beverages | 7224 |
| Restaurants and other food services | 722Z |
| Car washes | 811192 |

Table A.3 Localized Industries and the Corresponding NAICS continued…

| Industry Name | NAICS |
| --- | --- |
| Automotive repair and maintenance | 8111Z |
| Electronic and precision equipment repair and maintenance | 8112 |
| Commercial and industrial machinery and equipment repair and maintenance | 8113 |
| Footwear and leather goods repair | 81143 |
| Personal and household goods repair and maintenance | 8114Z |
| Barbershops | 812111 |
| Beauty salons | 812112 |
| Nail salons and other personal care services | 8121M |
| Funeral homes, cemeteries and crematories | 8122 |
| Dry-cleaning and laundry services | 8123 |
| Other personal services | 8129 |
| Religious organizations | 8131 |
| Labor unions | 81393 |
| Business, professional, political and similar organizations | 8139Z |
| Civic, social, advocacy organizations and grantmaking and giving services | 813M |
| Private households | 814 |

Table A.4 Traded Industries and the Corresponding NAICS

| Industry Name | NAICS |
| --- | --- |
| Crop production | 111 |
| Animal production | 112 |
| Logging | 1133 |
| Forestry except logging | 113M |
| Fishing, hunting, and trapping | 114 |
| Support activities for agriculture and forestry | 115 |
| Oil and gas extraction | 211 |
| Coal mining | 2121 |
| Metal ore mining | 2122 |
| Nonmetallic mineral mining and quarrying | 2123 |
| Support activities for mining | 213 |
| Electric power generation, transmission and distribution | 2211P |
| Water, steam, air conditioning, and irrigation systems | 2213M |
| Electric and gas, and other combinations | 221MP |
| Not specified utilities | 22S |
| Sugar and confectionery products | 3113 |
| Fruit and vegetable preserving and specialty foods | 3114 |
| Dairy products | 3115 |
| Animal slaughtering and processing | 3116 |
| Bakeries, except retail | 3118Z |
| Animal food, grain and oilseed milling | 311M1 |
| Seafood and other miscellaneous foods, n.e.c. | 311M2 |

Table A.4 Traded Industries and the Corresponding NAICS continued…

| Industry Name | NAICS |
| --- | --- |
| Not specified food industries | 311S |
| Beverage | 3121 |
| Tobacco | 3122 |
| Fiber, yarn, and thread mills | 3131 |
| Fabric mills, except knitting | 3132Z |
| Textile and fabric finishing and coating mills | 3133 |
| Carpets and rugs | 31411 |
| Textile product mills except carpets and rugs | 314Z |
| Cut and sew apparel | 3152 |
| Apparel accessories and other apparel | 3159 |
| Footwear | 3162 |
| Leather tanning and products, except footwear | 316M |
| Knitting mills | 31M |
| Sawmills and wood preservation | 3211 |
| Veneer, plywood, and engineered wood products | 3212 |
| Prefabricated wood buildings and mobile homes | 32199M |
| Miscellaneous wood products | 3219ZM |
| Pulp, paper, and paperboard mills | 3221 |
| Paperboard containers and boxes | 32221 |
| Miscellaneous paper and pulp products | 3222M |
| Printing and related support activities | 323 |
| Petroleum refining | 32411 |
| Miscellaneous petroleum and coal products | 3241M |
| Resin, synthetic rubber and fibers, and filaments | 3252 |
| Agricultural chemicals | 3253 |
| Pharmaceuticals and medicines | 3254 |
| Paint, coating, and adhesives | 3255 |
| Soap, cleaning compound, and cosmetics | 3256 |
| Industrial and miscellaneous chemicals | 325M |
| Plastics products | 3261 |
| Tires | 32621 |
| Rubber products, except tires | 3262M |
| Pottery, ceramics, and related products | 32711 |
| Structural clay products | 32712 |
| Glass and glass products | 3272 |
| Miscellaneous nonmetallic mineral products | 3279 |
| Cement, concrete, lime, and gypsum products | 327M |
| Aluminum production and processing | 3313 |
| Nonferrous metal, except aluminum, production and processing | 3314 |
| Foundries | 3315 |
| Iron and steel mills and steel products | 331M |
| Metal forgings and stampings | 3321 |

Table A.4 Traded Industries and the Corresponding NAICS continued…

| Industry Name | NAICS |
| --- | --- |
| Cutlery and hand tools | 3322 |
| Machine shops; turned products; screws, nuts and bolts | 3327 |
| Coating, engraving, heat treating and allied activities | 3328 |
| Ordnance | 33299M |
| Structural metals, and tank and shipping containers | 332M |
| Agricultural implements | 33311 |
| Construction mining and oil field machinery | 3331M |
| Commercial and service industry machinery | 3333 |
| Metalworking machinery | 3335 |
| Engines, turbines, and power transmission equipment | 3336 |
| Machinery, n.e.c. | 333M |
| Not specified machinery | 333S |
| Computer and peripheral equipment | 3341 |
| Navigational, measuring, electromedical, and control instruments | 3345 |
| Communications, audio, and video equipment | 334M1 |
| Electronic components and products, n.e.c. | 334M2 |
| Household appliances | 3352 |
| Electrical machinery, equipment, and supplies, n.e.c. | 335M |
| Aircraft and parts | 33641M1 |
| Aerospace products and parts | 33641M2 |
| Railroad rolling stock | 3365 |
| Ship and boat building | 3366 |
| Other transportation equipment | 3369 |
| Motor vehicles and motor vehicle equipment | 336M |
| Furniture and fixtures | 337 |
| Medical equipment and supplies | 3391 |
| Toys, amusement, and sporting goods | 3399M |
| Miscellaneous manufacturing, n.e.c. | 3399ZM |
| Not specified metal industries | 33MS |
| Not specified industries | 3MS |
| Furniture and home furnishing | 4232 |
| Professional and commercial equipment and supplies | 4234 |
| Metals and minerals, except petroleum | 4235 |
| Electrical goods | 4236 |
| Machinery, equipment, and supplies | 4238 |
| Miscellaneous durable goods | 4239Z |
| Paper and paper products | 4241 |
| Apparel, fabrics, and notions | 4243 |
| Farm product raw materials | 4245 |
| Petroleum and petroleum products | 4247 |
| Alcoholic beverages | 4248 |
| Farm supplies | 42491 |

Table A.4 Traded Industries and the Corresponding NAICS continued…

| Industry Name | NAICS |
| --- | --- |
| Miscellaneous nondurable goods | 4249Z |
| Drugs, sundries, and chemical and allied products | 424M |
| Not specified trade | 42S |
| Electronic auctions | 454112 |
| Air transportation | 481 |
| Rail transportation | 482 |
| Water transportation | 483 |
| Taxi and limousine service | 4853 |
| Pipeline transportation | 486 |
| Scenic and sightseeing transportation | 487 |
| Services incidental to transportation | 488 |
| Postal service | 491 |
| Warehousing and storage | 493 |
| Publishing, except newspapers and software | 5111Z |
| Software publishing | 5112 |
| Motion pictures and video industries | 5121 |
| Sound recording industries | 5122 |
| Other telecommunication services | 517Z |
| Libraries and archives | 51912 |
| Other information services | 5191Z |
| Data processing services | 5142 |
| Savings institutions, including credit unions | 5221M |
| Non-depository credit and related activities | 522M |
| Securities, commodities, funds, trusts, and other financial investments | 52M2 |
| Commercial, industrial, and other intangible assets rental and leasing | 53M |
| Architectural, engineering, and related services | 5413 |
| Specialized design services | 5414 |
| Computer systems design and related services | 5415 |
| Management, scientific and technical consulting services | 5416 |
| Scientific research and development services | 5417 |
| Advertising and related services | 5418 |
| Other professional, scientific and technical services | 5419Z |
| Management of companies and enterprises | 551 |
| Travel arrangements and reservation services | 5615 |
| Other administrative, and other support services | 561M |
| Colleges, including junior colleges, and universities | 611M1 |
| Business, technical, and trade schools and training | 611M2 |
| Other schools, instruction and educational services | 611M3 |
| Independent artists, performing arts, spectator sports and related industries | 711 |
| Museums, art galleries, historical sites, and similar institutions | 712 |
| Other amusement, gambling, and recreation industries | 713Z |
| Recreational vehicle parks and camps, and rooming and boarding houses | 721M |

Table A.5 Regression Results with Limited Years

|  | (1) | (2) | (3) | (4) |
| --- | --- | --- | --- | --- |
| VARIABLES | Market1 | Market2 | Market3 | Market4 |
|  |  |  |  |  |
| ${Treat}_{i}\times Post_{t}$ | -0.148** | 0.002 | -0.199 | -0.216 |
|  | (0.065) | (0.132) | (0.264) | (0.166) |
|  |  |  |  |  |
| Observations | 2,688 | 2,688 | 2,688 | 2,688 |
| Number of Puma | 672 | 672 | 672 | 672 |
| Adjusted R-squared | 0.183 | -0.240 | -0.199 | 0.023 |
| Controls | YES | YES | YES | YES |
| PUMA FE | YES | YES | YES | YES |
| Cluster SE | YES | YES | YES | YES |
| Time FE | YES | YES | YES | YES |

***Notes:*** *Standard errors in parentheses *** p<0.01, ** p<0.05, * p<0.1*

*The four labor market categories are: low-skill localized (Market1), low-skill tradable (Market2), high-skill localized (Market3), and high-skill tradable (Market4).*
